# Circular RNAs as Prognostic Biomarkers in Renal Cell Carcinoma: A Systematic Review and Meta-Analysis

**DOI:** 10.3389/fgene.2022.878700

**Published:** 2022-06-08

**Authors:** Dan Liao, Qiu Lin, Huan Xiao, Fenglian Zhang, Qin Han

**Affiliations:** ^1^ Department of Nephrology, Mianyang Central Hospital, School of Medicine, University of Electronic Science and Technology of China, Mianyang, China; ^2^ Department of Radiology, Mianyang Central Hospital, School of Medicine, University of Electronic Science and Technology of China, Mianyang, China; ^3^ School of Public Health, Chengdu Medical College, Chengdu, China

**Keywords:** circular RNAs, renal cell carcinoma, prognostic biomarkers, systematic review, meta-analysis

## Abstract

**Background:** Recently, several studies have shown that circRNAs play critical roles in renal cell carcinoma (RCC) oncogenesis and development. However, whether the level of circRNA expression in RCC is correlated with prognosis remains unclear. Hence, we conducted a meta-analysis to explore the association between circRNA expression levels and the prognosis of RCC patients.

**Methods:** We systematically searched Ovid, Embase, PubMed, and Web of Science from January 1950 to June 2021 for the literature published in English. According to the Preferred Reporting Items for Systematic Reviews and Meta-Analysis (PRISMA) guidelines, we conducted a meta-analysis of 21 selected studies to confirm the association between the circRNA expression level and prognosis of RCC.

**Results:** This meta-analysis included 20 articles and 1,559 RCC patients. The results showed that the high expression of oncogenic circRNAs (OS: HR = 2.04, 95% CI: 1.63–2.56, *p* = 0.20; PFS: HR = 2.82, 95% CI: 0.82–9.72, *p* = 0.03) and low expression of tumor-suppressor circRNAs (OS: HR: 1.92, 95% CI: 1.61–2.30, *p* < 0.05; PFS: HR: 2.40, 95% CI: 1.76–3.28, *p* = 0.36) were closely related to poor survival outcomes.

**Conclusion:** The meta-analysis verifies that circRNAs can be potential prognostic biomarkers of RCC.

## Introduction

Worldwide, kidney cancer has become a serious and widespread prevalent problem and is the 16th most frequently diagnosed cancer with the 17th highest mortality, accounting for 2.2% of all oncological diagnoses and 1.8% of all oncological deaths (Capitanio et al., 2019; Sung et al., 2021). Renal cell carcinoma (RCC) is a common cancer that originates in the renal epithelium and accounts for 90% of kidney cancers (Hsieh et al., 2017). Although the diagnosis and treatment (immunotherapy (Xu et al., 2020), targeted agents (Yang and Chen, 2020), and combination therapy (Cerbone et al., 2020)) of RCC have improved in the last 20 years, the overall survival of patients with RCC is still less than satisfactory (Haddad and Margulis, 2015; Xu et al., 2020). To guide clinical decision-making, a great prognostic evaluation of RCC is urgently necessary for both physicians and patients in treatment management. Currently, aside from imaging examination, reliable biomarkers that can be applied in clinical practice are lacking. Overall, more sensitive prognostic biomarkers and more effective therapeutic strategies for cancer need to be found.

CircRNAs are characterized by covalently closed-loop structures with neither 5′–3′ polarity nor polyadenylated tails (Chen and Yang, 2015). CircRNAs were identified as critical molecules in transcriptional regulation, splicing alternatives, interactions with RNA-binding proteins, and microRNA sponges in cellular physiology and disease pathogenesis (Han et al., 2018). CircRNAs have been confirmed as regulators and biomarkers for numerous types of cancers, which can act as either oncogenic or tumor suppressors in cancer and have also been shown to be enriched and stable in extracellular fluid (Chen and Huang, 2018). These findings indicate the potential of circRNAs to be effective biomarkers. Furthermore, as previously reported, circRNAs have a critical role in promoting metastasis in RCC (Yang et al., 2021). CircRNAs can contribute to tumorigenesis in RCC and promote proliferation and differentiation of RCC by regulating tumor-related signaling pathways (Li et al., 2020a) and activating transcription factors (Chen et al., 2020a). Thus, we reasonably predict that circRNAs may be potential effective therapeutic targets. In addition, many studies have identified that different expression levels of circRNAs are associated with survival in RCC patients. We therefore performed a meta-analysis to evaluate the prognostic value of circRNAs in RCC.

## Materials and Methods

### Literature Search

We searched the English medical literature in PubMed, Ovid, Embase, and Web of Science to identify all publications on circRNAs as prognostic biomarkers in human renal cell carcinoma. The database surveys were conducted on June 4, 2021. The following keywords were used in the database search: (“Circular RNA” and “Renal Cell Cancer”) (the detailed search terms are listed in Supplementary Table S1). We eliminated all irrelevant literature works by scanning the article title and abstract. We excluded all duplicated publications by using EndNote X9. The selected studies were identified after they were read in full by our reviewers.

### Publication Inclusion and Exclusion Criteria

The two investigators (DL and QH) independently used the same multistep process to evaluate whether these studies were suitable for our meta-analysis. A third investigator (QL) resolved any disagreements.

The inclusion criteria for meta-analysis followed the population, intervention, control, and outcome (PICO) criteria: *1*) patients with a pathological diagnosis of RCC, *2*) the expression of circRNAs in the tissue specimens of patients was measured, and *3*) the included studies provided time-to-event data, including overall survival (OS), progression-free survival (PFS), recurrence-free survival (RFS), disease-free survival (DFS), and association with circRNA expression. Considering the similar survival outcomes, RFS and DFS were combined as PFS (Zhou et al., 2015).

Furthermore, the excluded articles were eliminated based on the following criteria: *1*) the publications that were not published in English; *2*) letters, reviews, expert opinions, case reports, conference articles, clinical guidelines, and meeting records; *3*) studies of patient sample size <30; *4*) duplicated studies; *5*) studies of cell lines or animals; and *6*) the survival data shown in the article were not sufficient to calculate the HR value.

### Data Extraction

The two investigators (DL and HX) independently extracted relevant data for meta-analysis, and a third investigator (FZ) resolved any disagreements. Finally, we extracted the following items: the first author’s name, publication year, publication journal, circRNA type, total number of patients, sex, country, and follow-up period. The prognostic endpoints included hazard ratios (HRs) and 95% confidence intervals (CIs), OS, DFS, RFS, and PFS. With only the survival curve provided, Engauge Digitizer version 12.1 (available at http://sourceforge.net/) was used to extract related data from the survival curve. According to the extracted data and method of Spotswood et al. (Spruance et al., 2004), the HR was calculated.

### Quality Assessment

Two researchers (DL and QH) independently assessed the quality of all selected studies according to the Newcastle–Ottawa Scale (NOS) method (Stang, 2010). The two investigators identified all differences through discussion and consensus. Studies with NOS scores ranging from zero to normal and NOS scores ≥ six were considered high quality.

### Statistical Analysis

Meta-analysis was performed using a *meta* package (V.4.18–2). To determine heterogeneity between several studies, we used the *I* ^2^ test and the chi-based Q-test to assess statistical heterogeneity. If *I* ^2^ was equal to or <50%, the heterogeneity between studies was not obvious, so we used a fixed-effect model and instead applied the random-effect model (Borenstein et al., 2010). Finally, publication bias was evaluated with a funnel plot (a two-sided *p* < 0.05 was considered to be statistically significant). All statistical analyses were carried out in R V.3.6.1 (R Foundation for Statistical Computing). *p* ≤ 0.05 was considered to be statistically significant.

## Results

### Research Results

**FIGURE 1:**
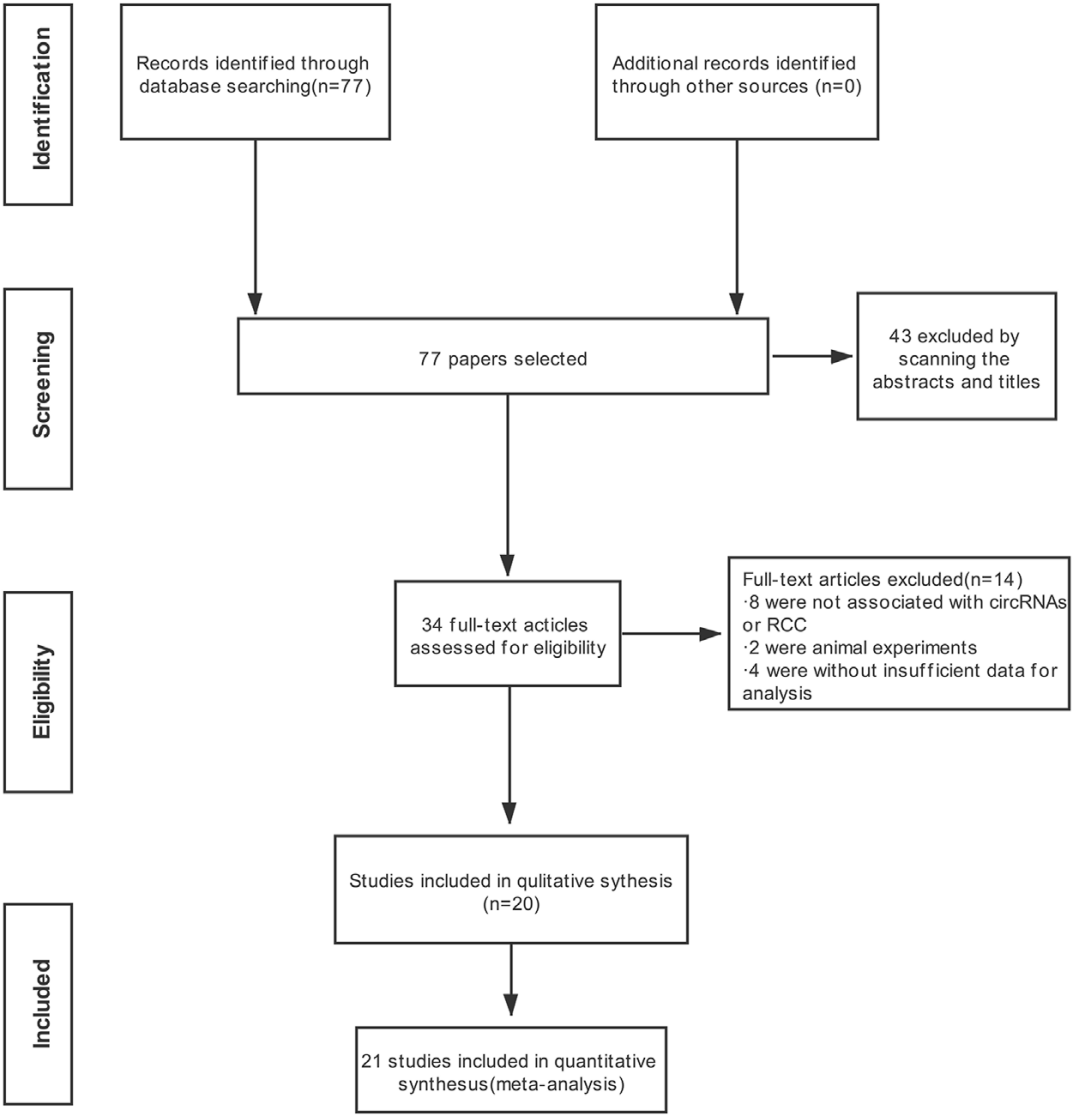
Flow chart demonstrating the study selection process.

Detailed information on the literature search is shown in Figure 1. A total of 77 publications in English were initially retrieved from the database. The most recent publication date was June 2021. In total, thirty-three articles were directly excluded after examining the abstract and title. For the remaining 34 publications, after careful reading, 14 studies were eliminated for the following reasons: eight were not associated with circRNAs or RCC, two were animal experiments, and four had insufficient data for analysis. A total of 21 studies were chosen for the meta-analysis based upon the inclusion and exclusion criteria.

### Study Characteristics and Quality Assessment

Of the 20 publications identified, 1,559 RCC patients were included, and the average number of patients was 77.95 (range 90.5–193.5). The main characteristics of the 10 studies are summarized in Table 1. One study was from Germany, two studies were from The Cancer Genome Atlas (TCGA) database, and other studies were from China. Together, 20 types of circRNAs were included in the meta-analysis (circHIAT1 (Wang et al., 2017; Wang et al., 2019), circNUP98 (Yu et al., 2020), circMYLK (Li et al., 2020a), hsa_circ_001895 (Chen et al., 2020a), circ‐EGLN3 (Lin and Cai, 2020), circ_001842 (Zeng et al., 2020), circ_0001368 (Chen et al., 2020b), cRAPGEF5 (Chen et al., 2020c), circ-ABCB10 (Huang et al., 2019), circCSNK1G3 (Li et al., 2021), circAKT1 (Zhu et al., 2020), circAGAP1 (Lv et al., 2021), circ_101341 (Yue et al., 2020), circEHD2 (Frey et al., 2021), circNETO2 (Frey et al., 2021), ciRS-7 (Zhao et al., 2020), hsa-hsa_circ_0085576 (Liu et al., 2020a), circTLK1 (Li et al., 2020b), circPTCH1 (Liu et al., 2020b), and circHIPK3 (Han et al., 2020)). Four circRNAs (circHIAT1, circ_0001368, cRAPGEF5, and circEHD2) were downregulated, and 16 circRNAs (circMYLK, hsa_circ_001895, circ-ABCB10, circAGAP1, circEHD2, hsa_circ_0085576, circTLK1, circPTCH1, circHIPK3, circNUP98, circ‐EGLN3, circ_001842, circCSNK1G3, circAKT1, and circ_101341) were upregulated.

**TABLE 1:** Characteristics of the included studies.

Study	Year	circRNA	No. of patients	Outcome	Expression regulation	Median OS	HR	Country	Multivariate analysis	Indirect
Kefeng Wang	2017	circHIAT1	40	OS	Downregulation	Low vs. high	3.75	China	No	Yes
Rui Yu	2020	circNUP98	65	OS	Upregulation	Low vs. high	0.48	China	No	Yes
Rui Yu	2020	circNUP98	65	DFS	Upregulation	Low vs. high	0.52	China	No	Yes
Jianfa Li	2020	circMYLK	71	OS	Upregulation	Low vs. high	1.25	China	No	Yes
Zhuangfei Chen	2019	hsa_circ_001895	60	OS	Upregulation	Low vs. high	0.63	China	No	Yes
Zhengmiao Wang	2019	circHIAT1	80	OS	Downregulation	Low vs. high.	1.90	China	No	Yes
Ling Lin	2019	circ‐EGLN3	80	OS	Upregulation	Low vs. high	0.59	China	No	Yes
Jiawei Zeng	2020	circ_001842	97	OS	Upregulation	Low vs. high	0.37	China	No	Yes
Lin Chen	2020	circ_0001368	64	OS	Downregulation	Low vs. high	1.92	China	Yes	No
Qiong Chen	2020	cRAPGEF5	245	OS	Downregulation	Low vs. high	1.79	China	Yes	No
Qiong Chen	2020	cRAPGEF5	245	RFS	Downregulation	Low vs. high	1.64	China	Yes	No
Yunfang Huang	2021	Circ-ABCB10	120	OS	Upregulation	High vs. low	5.29	China	Yes	No
Wen Li	2020	circCSNK1G3	64	OS	Upregulation	Low vs. high	0.34	TCGA-KICH	No	Yes
Qingliang Zhu	2021	CircAKT1	70	OS	Upregulation	Low vs. high	0.56	China	No	Yes
Qi Lv	2021	CircAGAP1		OS	Upregulation	High vs. low	1.68	TCGA-KICH	No	No
Yongjun Yue	2020	Circ_101341	60	OS	Upregulation	High vs. low	0.42	China	No	Yes
Lisa Frey	2021	circEHD2	121	PFS	Downregulation	High vs. low	3.58	Germany	Yes	No
Lisa Frey	2021	circNETO2	121	PFS	Upregulation	High vs. low	0.17	Germany	Yes	No
Lisa Frey	2021	circEHD2	121	CSS	Downregulation	High vs. low	2.67	Germany	Yes	No
Lisa Frey	2021	circNETO2	121	CSS	Upregulation	High vs. low	0.14	Germany	Yes	No
Lisa Frey	2021	circEHD2	121	OS	Downregulation	High vs. low	3.91	Germany	Yes	No
Lisa Frey	2021	circNETO2	121	OS	Upregulation	High vs. low	0.15	Germany	Yes	No
Yanhui Zhao	2020	ciRS-7	87	PFS	Upregulation	Low vs. high	0.53	China	No	Yes
Guanghua Liu	2020	has-hsa_circ_0085576	86	OS	Upregulation	High vs. low	1.37	China	Yes	No
Guanghua Liu	2020	has-hsa_circ_0085576	86	DFS	Upregulation	High vs. low	2.14	China	No	Yes
Jianfa Li	2020	CircTLK1	60	OS	Upregulation	Low vs. high	0.45	China	No	Yes
Jianfa Li	2020	CircTLK1	60	DFS	Upregulation	Low vs. high	0.86	China	No	Yes
Huan Liu	2020	circPTCH1	39	OS	Upregulation	Low vs. high	0.65	China	No	Yes
Bin Han	2020	CircHIPK3	50	OS	Upregulation	Low vs. high	0.42	China	No	Yes

PS: indirect: we indirectly calculated HR from the plot; multivariate analysis: multivariate analysis was used to adjust HR.

The NOS for quality evaluation of the included studies varied from 6 to 8, indicating that all included studies were available and were high-quality studies. Among the 20 identified articles, NOS assessment included 4 documents with an NOS score of 6, 6 articles with an NOS score of 7, and 10 articles with an NOS score of 8. The value of the k-statistic was 0.87, indicating excellent agreement between the two reviewers. Therefore, all 20 eligible studies underwent meta-analysis (Table 2).

**TABLE 2:** Quality assessment was based on the Newcastle–Ottawa Scale (NOS).

First author	Year	Selection	Comparability	Outcome	Total score
Wang et al. (2017)	2017	4	2	1	7
Yu et al. (2020)	2020	3	2	1	6
Li et al. (2020a)	2020	4	2	1	7
Chen et al. (2020a)	2019	4	2	2	8
Wang et al. (2019)	2019	3	2	2	7
Lin and Cai (2020)	2019	4	1	1	6
Zeng et al. (2020)	2020	4	2	1	7
Chen et al. (2020b)	2020	4	2	2	8
Chen et al. (2020c)	2020	3	2	2	7
Huang et al. (2019)	2019	4	1	1	6
Li et al. (2021)	2020	3	2	2	7
Zhu et al. (2020)	2020	4	2	2	8
Lv et al. (2021)	2021	4	2	2	8
Yue et al. (2020)	2020	3	1	2	6
Frey et al. (2021)	2021	4	1	1	6
Zhao et al. (2020)	2020	4	2	2	8
Liu et al. (2020a)	2020	3	2	2	7
Li et al. (2020b)	2020	4	2	1	7
Liu et al. (2020b)	2020	4	1	2	7
Han et al. (2020)	2020	4	2	2	8

### The Relationship Between circRNA Expression and Survival Outcomes

#### The Relationship Between circRNA Expression and OS

**FIGURE 2:**
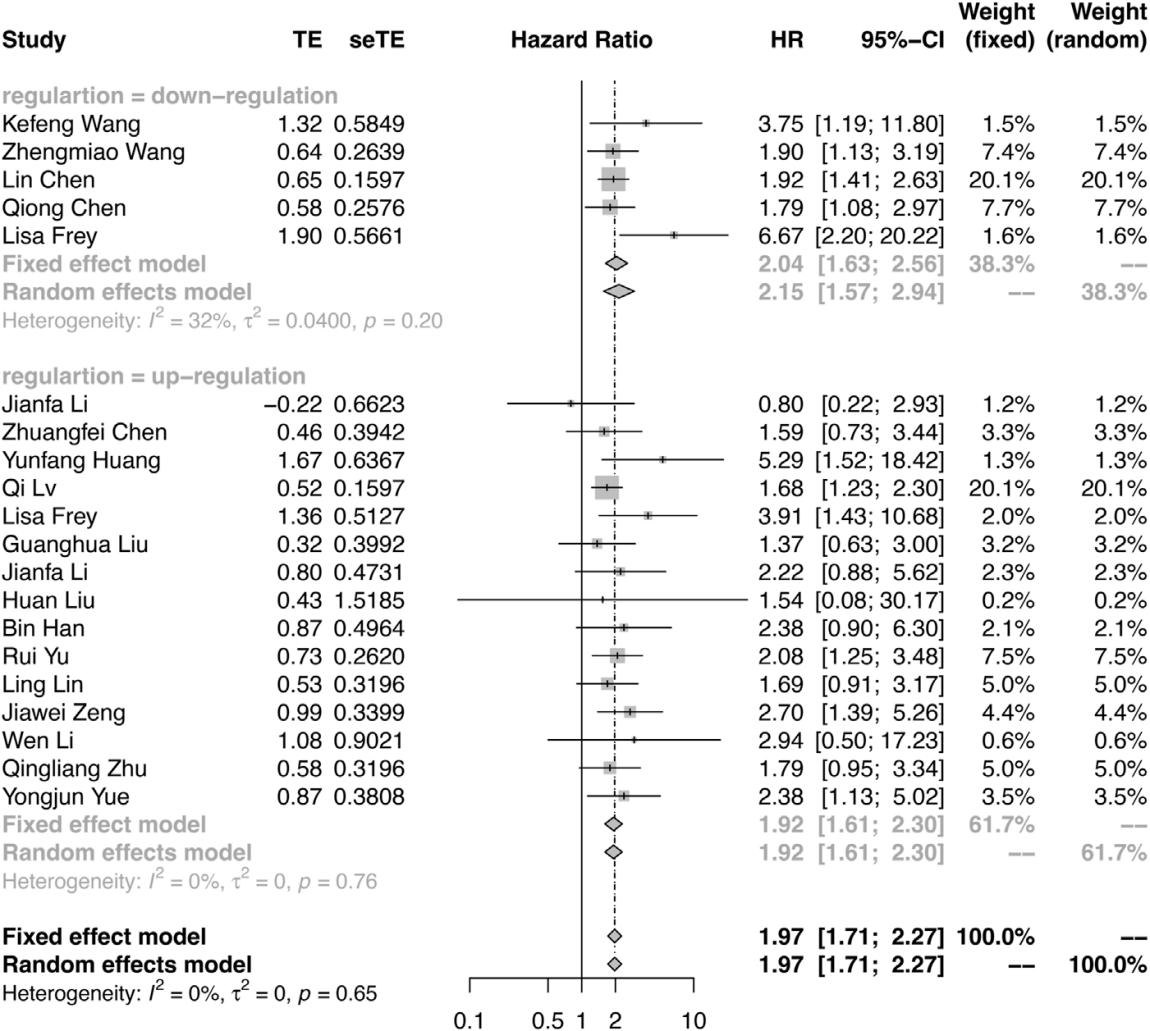
Forest plots verify the association between the expression of circRNAs and overall survival (OS). High expression of oncogenic circRNAs and low expression of tumor-suppressor circRNAs were associated with poor OS.

The meta-analysis defined OS as the primary endpoint. Among the 20 publications, all studies verified the relationship between OS and circRNA expression levels (Supplementary Table S2). As shown in Figure 2, because of the few heterogeneities among the included studies (OS: downregulation: I^2^ = 32%, *p* = 0.2; upregulation: I^2^ = 0%, *p* = 0.75), the pooled HR and corresponding 95% CI were estimated by applying the fixed-effect model. Overall, the results demonstrated that the differential expression of circRNAs is closely related to poor survival in RCC (HR = 1.97, 95% CI: 1.71–2.27, *p* < 0.0001). The results indicated that patients with oncogenic circRNA overexpression had worse OS than those with low expression (OS: HR = 2.04, 95% CI: 1.63–2.56, *p* < 0.0001). Furthermore, the results demonstrated that the lower expression of some tumor-suppressor circRNAs was associated with poor OS (HR: 1.92, 95% CI: 1.61–2.30, *p* < 0.0001) (Figure 2).

### The Relationship Between circRNA Expression and PFS

**FIGURE 3:**
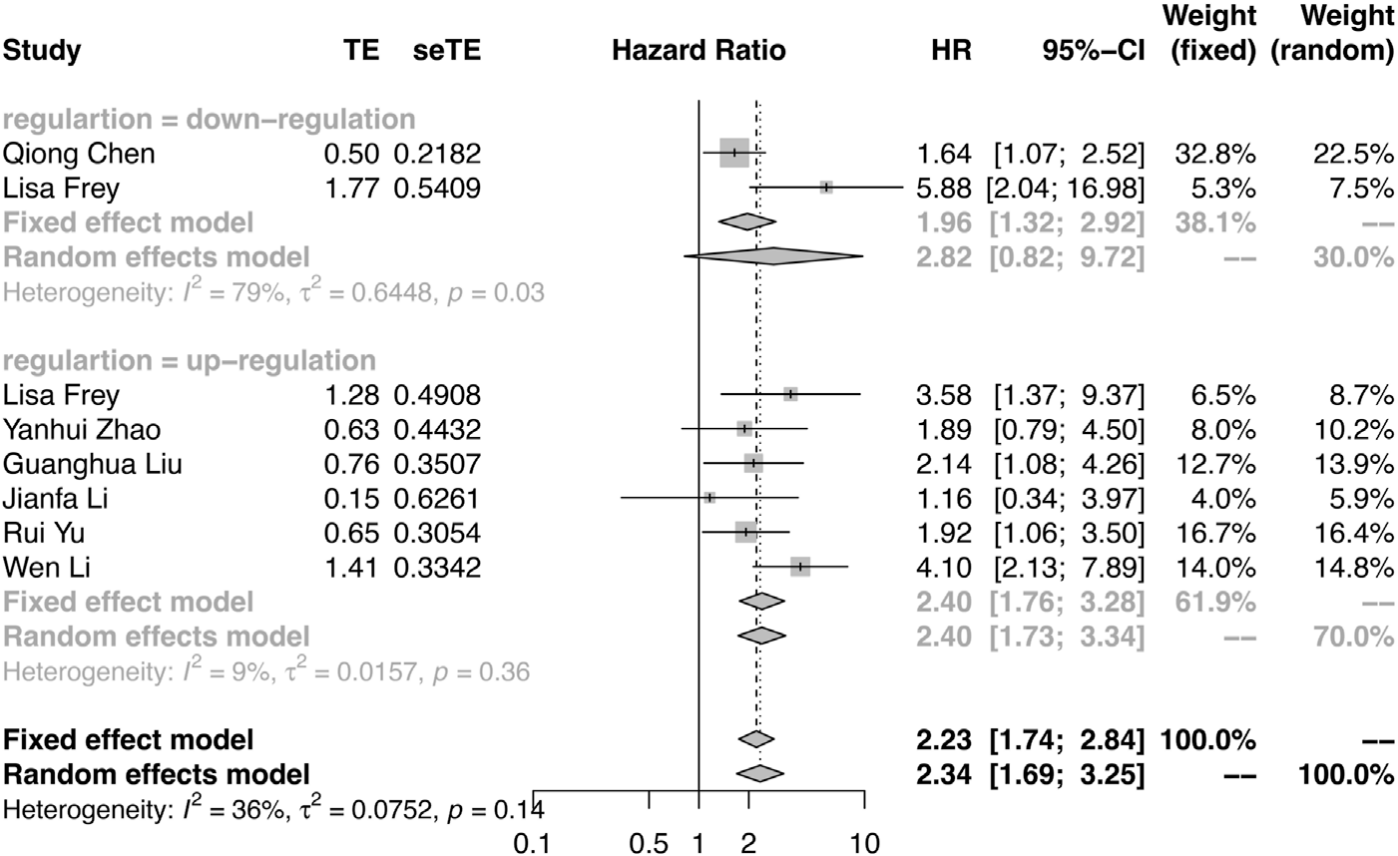
Forest plots verify the association between the expression of circRNAs and progression-free survival (PFS). High expression of oncogenic circRNAs and low expression of tumor-suppressor circRNAs were associated with poor PFS.

Among the included studies, PFS was reported in three studies, RFS was reported in one study, and DFS was reported in four studies (Supplementary Table S3). Considering the similar survival outcomes, RFS and DFS were combined as PFS. As shown in Figure 3, there were large heterogeneities (I^2^ = 79%, *p* = 0.03) in the downregulation group and a few heterogeneities (I^2^ = 9%, *p* = 0.36) in the upregulation group. A random effect model was applied in the downregulation group, and a fixed effect model was applied in the upregulation group. The results also demonstrated that the oncogenic circRNA overexpression was associated with worse PFS than low expression (PFS: HR = 2.82, 95% CI: 0.82–9.72, *p* < 0.0001), and lower expression of tumor-suppressor circRNAs was correlated with poor PFS (HR: 2.40, 95% CI: 1.76–3.28, *p* = 0.0009) (Figure 3).

#### Meta-Regression

Meta-regression was performed based on OS. We performed the univariate meta-regression and multivariate meta-regression to further assess the heterogeneity (Table 3). The results revealed that plot and country of multivariate analysis may significantly influence the variation in HR (univariate meta-regression: *p*-value for year = 0.659, *p*-value for expression regulation = 0.682, *p*-value for indirect = 0.768, *p*-value for multivariate analysis = 0.531, and *p*-value for country; multivariate meta-regression: *p*-value for year = 0.611, *p*-value for regulation = 0.966, *p*-value for indirect = 0.019, *p*-value for multivariate analysis = 0.013, and *p*-value for country = 0.016).

**TABLE 3:** Meta-regression analysis of the included studies.

**Factor**	**Univariate meta-regression *p*-value**	**95% CI**
Year	0.659	−0.2339 to 0.1479
Regulation	0.682	−0.3491 to 0.9419
Direct vs. indirect	0.768	−0.2401 to 0.3253
Multi	0.531	−0.1992 to 0.3861
Country	0.912	−0.3458 to 0.3087
**Factor**	**Multivariate meta-regression analysis *p*-value**	**95% CI**
Year	0.611	−0.4241 to 0.2495
Regulation	0.966	−0.4606 to 0.4413
Indirect	0.019	0.1613 to 1.8256
Multi	0.013	0.2048 to 1.7720
Country	0.016	−1.7281 to −0.1788

PS: 1) Year: publication year; 2) Regulation: upregulation vs. downregulation; 3) Direct: extract HR from the manuscript, indirect: calculate HR from the plot; 4) Multivariate analysis: multivariate analysis was used to adjust HR; 5) Country: China vs. TCGA or Germany.

### Publication Bias

**FIGURE 4:**
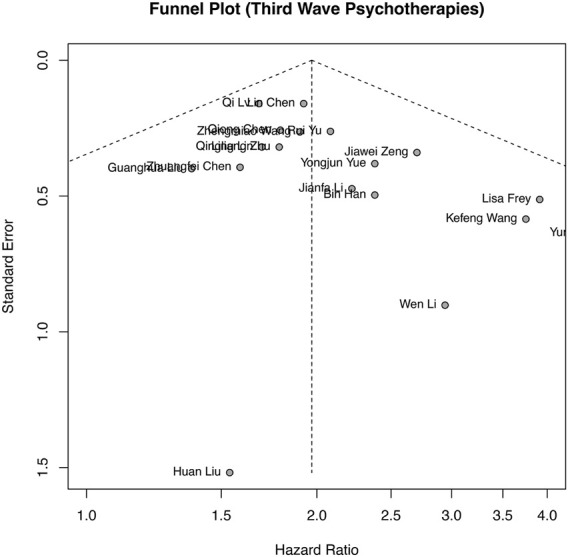
Funnel plots of studies included in the meta-analyses.

Publication bias analysis was performed based on OS, as shown in Figure 4, and the funnel plot was approximately symmetrical. Publication bias was also assessed by Begg’s and Egger’s tests in the meta-analysis. The results (Begg’s *p* = 0.0693 and Egger’s *p* = 0.0798) and funnel plot all indicated no obvious publication bias among the included studies.

## Discussion

RCC is characterized by both high mortality and morbidity (Jonasch et al., 2014). Metastasis and recurrence are some of the leading causes of death. Therefore, more accurate biomarkers are needed for predicting the prognosis of RCC patients to monitor the patient’s condition. Here, we summarized the prognostic value of circRNAs in RCC. This meta-analysis, including 21 studies and 1,559 RCC patients, is the first to investigate the relationship between circRNAs and the prognosis of RCC patients. According to the included studies, we found that oncogenic circRNAs with high expression and tumor-suppressor circRNAs with lower expression were correlated with worse survival, which indicated that circRNAs may play important roles in tumor initiation and progression.

CircRNAs play both oncogenic and tumor-suppressor roles in RCC (Yang et al., 2021). CircRNAs function predominantly by acting as sponges of microRNAs. In this way, circRNAs can regulate tumor-related signaling pathways. Li et al. (2020a) found that circMYLK is notably upregulated in RCC and that circMYLK upregulation can promote tumor growth. This role was achieved by circular RNA MYLK regulating miR-513a-5p/VEGFC signaling. Furthermore, Zhang et al. also found that circular RNA hsa_circ_0054537 can regulate the cMet pathway to promote the progression of RCC by sponging miR-130a-3p (Li et al., 2020c). In addition to its oncogenic role, Chen et al. (2019) also found that circular RNA hsa-circ-0072309 can play an antitumor role by deactivating the PI3K/AKT and mTOR pathways by sponging miR-100. Sun et al. (2020) found that circUBAP2 can regulate the miR-148a-3p/FOXK2 pathway to inhibit the proliferation and metastasis of RCC. Furthermore, circRNAs can also regulate transcription factors to impact tumor initiation and evolution. For example, circular RNA hsa_circ_001895 regulates SRY-box transcription factor 12 (SOX12) by sponging microRNA-296-5p to promote RCC progression (Chen et al., 2020a). These studies indicate that circRNAs play an important role in tumorigenesis, tumor development, and metastasis, which demonstrates that circRNAs have the potential to act as biomarkers for the prognosis of RCC.

However, we tried to ensure the authenticity and reliability of the meta-analysis. Nonetheless, there are still some limitations in this research. Only 1,559 RCC patients were included, and all samples were from China and Germany, which also leads to publication bias. Thus, more studies performed in other parts of the world are needed. Furthermore, because some HR values were not reported in the article, HR was calculated by Kaplan/Meier curves in some studies lacking HR values. Subjective factors may be introduced. Future studies investigating the relationship between the expression of circRNAs and prognosis in RCC need to provide more complete data.

In summary, we performed a meta-analysis to identify the prognostic value of circRNAs in RCC patients. The results demonstrate that the high expression of circRNAs with cancer-promoting effects and the low expression of circRNAs with tumor-suppressing effects are associated with poor prognosis in RCC patients. Furthermore, many circRNAs play significant roles in RCC initiation and progression. Future studies utilizing circRNAs may demonstrate an effective prognostic biomarker in all RCC patients.

## Data Availability

The original contributions presented in the study are included in the article/Supplementary Materials; further inquiries can be directed to the corresponding author.

## Abbreviations

circRNAs, circular RNAs; DFS, disease-free survival; HRs, hazard ratios; NOS, Newcastle–Ottawa Scale; OS, overall survival; PFS, progression-free survival; RCC, renal cell carcinoma; RFS, relapse-free survival.

## References

[B1] Borenstein M. Hedges L. V. Higgins J. P. T. Rothstein H. R. (2010). A Basic Introduction to Fixed-Effect and Random-Effects Models for Meta-Analysis. Res. Synth. Method 1 (2), 97–111. 10.1002/jrsm.12 26061376

[B2] Capitanio U. Bensalah K. Bex A. Boorjian S. A. Bray F. Coleman J. (2019). Epidemiology of Renal Cell Carcinoma. Eur. Urol. 75 (1), 74–84. 10.1016/j.eururo.2018.08.036 30243799 PMC8397918

[B3] Cerbone L. Cattrini C. Vallome G. Latocca M. M. Boccardo F. Zanardi E. (2020). Combination Therapy in Metastatic Renal Cell Carcinoma: Back to the Future? Seminars Oncol. 47 (6), 361–366. 10.1053/j.seminoncol.2020.10.003 33168323

[B4] Chen B. Huang S. (2018). Circular RNA: An Emerging Non-coding RNA as a Regulator and Biomarker in Cancer. Cancer Lett. 418, 41–50. 10.1016/j.canlet.2018.01.011 29330104

[B5] Chen L.-L. Yang L. (2015). Regulation of circRNA Biogenesis. RNA Biol. 12 (4), 381–388. 10.1080/15476286.2015.1020271 25746834 PMC4615371

[B6] Chen L. Wu D. Ding T. (2020). Circular RNA Circ_0001368 Inhibited Growth and Invasion in Renal Cell Carcinoma by Sponging miR-492 and Targeting LATS2. Gene 753, 144781. 10.1016/j.gene.2020.144781 32428698

[B7] Chen Q. Liu T. Bao Y. Zhao T. Wang J. Wang H. (2020). CircRNA cRAPGEF5 Inhibits the Growth and Metastasis of Renal Cell Carcinoma via the miR-27a-3p/TXNIP Pathway. Cancer Lett. 469, 68–77. 10.1016/j.canlet.2019.10.017 31629934

[B8] Chen T. Shao S. Li W. Liu Y. Cao Y. (2019). RETRACTED ARTICLE: The Circular RNA Hsa-Circ-0072309 Plays Anti-tumour Roles by Sponging miR-100 through the Deactivation of PI3K/AKT and mTOR Pathways in the Renal Carcinoma Cell Lines. Artif. Cells, Nanomedicine, Biotechnol. 47 (1), 3638–3648. 10.1080/21691401.2019.1657873 31456425

[B9] Chen Z. Xiao K. Chen S. Huang Z. Ye Y. Chen T. (2020). Circular RNA Hsa_circ_001895 Serves as a Sponge of microRNA‐296‐5p to Promote Clear Cell Renal Cell Carcinoma Progression by Regulating SOX12. Cancer Sci. 111 (2), 713–726. 10.1111/cas.14261 31782868 PMC7004537

[B10] Frey L. Klümper N. Schmidt D. Kristiansen G. Toma M. Ritter M. (2021). CircEHD2, CircNETO2 and CircEGLN3 as Diagnostic and Prognostic Biomarkers for Patients with Renal Cell Carcinoma. Cancers 13 (9), 2177. 10.3390/cancers13092177 33946584 PMC8124893

[B11] Haddad A. Q. Margulis V. (2015). Tumour and Patient Factors in Renal Cell Carcinoma-Towards Personalized Therapy. Nat. Rev. Urol. 12 (5), 253–262. 10.1038/nrurol.2015.71 25868564

[B12] Han B. Chao J. Yao H. (2018). Circular RNA and its Mechanisms in Disease: From the Bench to the Clinic. Pharmacol. Ther. 187, 31–44. 10.1016/j.pharmthera.2018.01.010 29406246

[B13] Han B. Shaolong E. Luan L. Li N. Liu X. (2020). CircHIPK3 Promotes Clear Cell Renal Cell Carcinoma (ccRCC) Cells Proliferation and Metastasis via Altering of miR-508-3p/CXCL13 Signal. Ott 13, 6051–6062. 10.2147/OTT.S251436 PMC7422843 32821115

[B14] Hsieh J. J. Purdue M. P. Signoretti S. Swanton C. Albiges L. Schmidinger M. (2017). Renal Cell Carcinoma. Nat. Rev. Dis. Prim. 3, 17009. 10.1038/nrdp.2017.9 28276433 PMC5936048

[B15] Huang Y. Zhang Y. Jia L. Liu C. Xu F. (2019). Circular RNA ABCB10 Promotes Tumor Progression and Correlates with Pejorative Prognosis in Clear Cell Renal Cell Carcinoma. Int. J. Biol. Markers 34 (2), 176–183. 10.1177/1724600819842279 31106654

[B16] Jonasch E. Gao J. Rathmell W. K. (2014). Renal Cell Carcinoma. BMJ 349, g4797. 10.1136/bmj.g4797 25385470 PMC4707715

[B17] Li J. Huang C. Zou Y. Ye J. Yu J. Gui Y. (2020). CircTLK1 Promotes the Proliferation and Metastasis of Renal Cell Carcinoma by Sponging miR-136-5p. Mol. Cancer 19 (1), 103. 10.1186/s12943-020-01225-2 32503552 PMC7275467

[B18] Li J. Huang C. Zou Y. Yu J. Gui Y. (2020). Circular RNA MYLK Promotes Tumour Growth and Metastasis via Modulating miR‐513a‐5p/VEGFC Signalling in Renal Cell Carcinoma. J. Cell. Mol. Med. 24 (12), 6609–6621. 10.1111/jcmm.15308 32342645 PMC7299689

[B19] Li R. Luo S. Zhang D. (2020). Circular RNA Hsa_circ_0054537 Sponges miR-130a-3p to Promote the Progression of Renal Cell Carcinoma through Regulating cMet Pathway. Gene 754, 144811. 10.1016/j.gene.2020.144811 32464246

[B20] Li W. Song Y. Y. Y. Rao T. Yu W. M. Ruan Y. Ning J. Z. (2021). CircCSNK1G3 Up‐regulates miR‐181b to Promote Growth and Metastasis via TIMP3‐mediated Epithelial to Mesenchymal Transitions in Renal Cell Carcinoma. J. Cell. Mol. Medi 26, 1729–1741. 10.1111/jcmm.15911 PMC8918408 33560588

[B21] Lin L. Cai J. (2020). Circular RNA circ‐EGLN3 Promotes Renal Cell Carcinoma Proliferation and Aggressiveness via miR‐1299‐mediated IRF7 Activation. J. Cell. Biochem. 121 (11), 4377–4385. 10.1002/jcb.29620 31904147

[B22] Liu G. Zhou J. Piao Y. Zhao X. Zuo Y. Ji Z. (2020). Hsa_circ_0085576 Promotes Clear Cell Renal Cell Carcinoma Tumorigenesis and Metastasis through the miR-498/YAP1 axis. Aging 12 (12), 11530–11549. 10.18632/aging.103300 32541093 PMC7343478

[B23] Liu H. Hu G. Wang Z. Liu Q. Zhang J. Chen Y. (2020). circPTCH1 Promotes Invasion and Metastasis in Renal Cell Carcinoma via Regulating miR-485-5p/MMP14 axis. Theranostics 10 (23), 10791–10807. 10.7150/thno.47239 32929380 PMC7482820

[B24] Lv Q. Wang G. Zhang Y. Shen A. Tang J. Sun Y. (2021). CircAGAP1 Promotes Tumor Progression by Sponging miR-15-5p in Clear Cell Renal Cell Carcinoma. J. Exp. Clin. Cancer Res. 40 (1), 76. 10.1186/s13046-021-01864-3 33618745 PMC7901094

[B25] Spruance S. L. Reid J. E. Grace M. Samore M. (2004). Hazard Ratio in Clinical Trials. Antimicrob. Agents Chemother. 48 (8), 2787–2792. 10.1128/aac.48.8.2787-2792.2004 15273082 PMC478551

[B26] Stang A. (2010). Critical Evaluation of the Newcastle-Ottawa Scale for the Assessment of the Quality of Nonrandomized Studies in Meta-Analyses. Eur. J. Epidemiol. 25 (9), 603–605. 10.1007/s10654-010-9491-z 20652370

[B27] Sun J. Yin A. Zhang W. Lv J. Liang Y. Li H. (2020). CircUBAP2 Inhibits Proliferation and Metastasis of Clear Cell Renal Cell Carcinoma via Targeting miR-148a-3p/FOXK2 Pathway. Cell. Transpl. 29, 096368972092575. 10.1177/0963689720925751 PMC7563813 32425115

[B28] Sung H. Ferlay J. Siegel R. L. Laversanne M. Soerjomataram I. Jemal A. (2021). Global Cancer Statistics 2020: GLOBOCAN Estimates of Incidence and Mortality Worldwide for 36 Cancers in 185 Countries. CA A Cancer J. Clin. 71 (3), 209–249. 10.3322/caac.21660 33538338

[B29] Wang K. Sun Y. Tao W. Fei X. Chang C. (2017). Androgen Receptor (AR) Promotes Clear Cell Renal Cell Carcinoma (ccRCC) Migration and Invasion via Altering the circHIAT1/miR-195-5p/29a-3p/29c-3p/CDC42 Signals. Cancer Lett. 394, 1–12. 10.1016/j.canlet.2016.12.036 28089832

[B30] Wang Z. Zhao Y. Wang Y. Jin C. (2019). Circular RNA circHIAT1 Inhibits Cell Growth in Hepatocellular Carcinoma by Regulating miR-3171/PTEN axis. Biomed. Pharmacother. 116, 108932. 10.1016/j.biopha.2019.108932 31108351

[B31] Xu W. Atkins M. B. McDermott D. F. (2020). Checkpoint Inhibitor Immunotherapy in Kidney Cancer. Nat. Rev. Urol. 17 (3), 137–150. 10.1038/s41585-020-0282-3 32020040

[B32] Yang D. C. Chen C.-H. (2020). Potential New Therapeutic Approaches for Renal Cell Carcinoma. Seminars Nephrol. 40 (1), 86–97. 10.1016/j.semnephrol.2019.12.010 32130970

[B33] Yang L. Zou X. Zou J. Zhang G. (2021). Functions of Circular RNAs in Bladder, Prostate and Renal Cell Cancer (Review). Mol. Med. Rep. 23 (5). 10.3892/mmr.2021.11946 PMC7974260 33649838

[B34] Yu R. Yao J. Ren Y. (2020). A Novel circRNA, circNUP98, a Potential Biomarker, Acted as an Oncogene via the miR‐567/PRDX3 axis in Renal Cell Carcinoma. J. Cell. Mol. Med. 24 (17), 10177–10188. 10.1111/jcmm.15629 32729669 PMC7520319

[B35] Yue Y. Cui J. Zhao Y. Liu S. Niu W. (2020). Circ_101341 Deteriorates the Progression of Clear Cell Renal Cell Carcinoma through the miR- 411/EGLN3 Axis. Cmar 12, 13513–13525. 10.2147/CMAR.S272287 PMC7781030 33408523

[B36] Zeng J. Feng Q. Wang Y. Xie G. Li Y. Yang Y. (2020). Circular RNA Circ_001842 Plays an Oncogenic Role in Renal Cell Carcinoma by Disrupting microRNA‐502‐5p‐mediated Inhibition of SLC39A14. J. Cell. Mol. Med. 24 (17), 9712–9725. 10.1111/jcmm.15529 32729666 PMC7520279

[B37] Zhao Y.-H. Wang Z. Zhang N. Cui T. Zhang Y.-H. (2020). Effect of ciRS-7 Expression on Clear Cell Renal Cell Carcinoma Progression. Chin. Med. J. Engl. 133 (17), 2084–2089. 10.1097/CM9.0000000000000867 32496306 PMC7478654

[B38] Zhou J.-G. Tian X. Wang X. Tian J.-H. Wang Y. Wang F. (2015). Treatment on Advanced NSCLC: Platinum-Based Chemotherapy Plus Erlotinib or Platinum-Based Chemotherapy Alone? A Systematic Review and Meta-Analysis of Randomised Controlled Trials. Med. Oncol. 32 (2), 471. 10.1007/s12032-014-0471-0 25579169

[B39] Zhu Q. Zhan D. Zhu P. Chong Y. Yang Y. (2020). CircAKT1 Acts as a Sponge of miR-338-3p to Facilitate Clear Cell Renal Cell Carcinoma Progression by Up-Regulating CAV1. Biochem. Biophysical Res. Commun. 532 (4), 584–590. 10.1016/j.bbrc.2020.08.081 32900491

